# Abstracts from the British Society of Echocardiography annual meeting 2023

**DOI:** 10.1186/s44156-024-00053-0

**Published:** 2024-07-04

## BSEcho 2023 conference report

It was a great pleasure for the British Society of Echocardiography to host delegates for their annual conference 2023 at the International Conference Centre Wales. The ICC Wales was the perfect venue with a spacious exhibition room and auditorium and quality rooms for the parallel streams all supported by the wonderful Welsh hospitality and catering. The format of delivery was developed from last year with the continuation of the hybrid format which gave all the members the opportunity to engage either through face-to-face or via the online platform with over 1200 delegates attending in total.

The conference was opened by our out-going president, Dr. Claire Colebourn, who provided the perfect starting point and platform for the subsequent enriched content from high quality, expert speakers delivered across parallel sessions. For the first time a track dedicated to congenital heart disease and inherited cardiomyopathies was provided on Friday, and intensivist echocardiography on Saturday. These sessions were sandwiched by an auditorium packed programme and the pre-accreditation/research and audit sessions. We were delighted to welcome our International speaker, Dr. Judy Hung, who provided us with a fantastic journey of echocardiography from historical perspectives to future directions, and our invited speaker, Prof. Sanjay Sharma, who entertained a full auditorium of keen delegates with his insights into the athletes heart; we were also entranced by the debate on artificial intelligence from the brains and wit of Professors Paul Leeson and Rick Steeds.

The educational content was supported by ongoing workshops that provided expertise insight on focused ‘hot topics’ including: musculoskeletal health, TOE, Stress Echocardiography and Diastolic function (providing practical insight into the earlier delivered proposed new BSE guidelines). These workshops were attended and taught through enthusiasm and interest underpinning the BSE’s widening philosophy for education. The exhibition room was a buzz of networking and engagement with the 30 exhibitors providing that important link to industry and service. It was also fantastic to see high quality research provided through posters and oral presentations and we would like to congratulate Eleanor Drew for her excellent study and presentation ‘Identification of a three-dimensional transthoracic echocardiography derived correction factor to improve the accuracy of left ventricular outflow tract measurements’ with her being a truly worthy recipient of the Investigator of the Year award.

Although this year was a resounding success and the echocardiographic community never felt as close, it was delivered shortly after the premature passing of our great echocardiographer and pioneer Professor Mark Monaghan. His life and his character were remembered throughout the conference and a dedicated evening meal was a fitting tribute to him, allowing colleagues to remember Mark and share stories of his life and contributions to the BSE.

The Annual General Meeting saw our new president, Prof Dan Augustine take the reins and we were pleased to see him present his member-focussed pledge that will take our Society onwards and upwards. We are now building and preparing for Edinburgh 2024. We are extremely excited that this will be another huge success and will continue to provide the high-quality education, the diverse content and the excellent space for shared ideas and practice. Until then let’s embrace our year in echocardiography.

**Professor Dave Oxborough and Dr. Liam Ring**

Co-Chairs of Education

ABS001

**Withdrawn**.

## ABS003 Identification of a three-dimensional transthoracic echocardiography derived correction factor to improve the accuracy of left ventricular outflow tract measurements

### Jane James^1^, Trystan John^1^, Badri Chandrasekaran^1^, Guilherme Artioli^2^, Eleanor Drew^1,2^

#### ^1^Great Western Hospital NHS Foundation Trust, Swindon, UK; ^2^Manchester Metropolitan University, UK

*Echo Research & Practice* 2024, **11(Suppl 1)**:ABS003

**Background:** The limitations that the left ventricular outflow tract (LVOT) is circular can result in underestimation of LVOT area and subsequently aortic valve area (AVA) when calculated from standard transthoracic echocardiograms (TTE). Three-dimensional (3D) techniques have allowed more precise LVOT measurements, eliminating geometric assumptions associated with two-dimensional (2D) imaging.

**Purpose/aims:** To determine the feasibility and reproducibility of 3D LVOT measurements. Secondly, to improve accuracy of 2D TTE-based AVA calculation by introducing a correction factor (CF) derived from 3D TTE images and validating this in aortic stenosis (AS) patients.

**Methods:** In this retrospective pilot study patients with any degree of AS had an LVOT diameter (LVOTd) measured from a 2D parasternal long axis image (D1). Additionally, a 3D zoom dataset of the LVOT, and aortic valve was obtained. 3D LVOT area planimetry could be performed in 93% of patients, and subsequently converted into a theoretical circle, and a diameter provided (D2). D2/D1 derived the CF. CF validation was via determining the effect on the correlation between AVA and haemodynamic AS parameters. Finally, the ability of the CF to reclassify AS severity was determined.

**Results:** D1 and D2 were significantly different (P < 0.001) and subsequently a CF of 1.1 was derived. Operator variability of 2D LVOTd was superior to 3D LVOT area planimetry. LVOT area was underestimated when calculated from 2D LVOTd, compared to LVOT area planimetry, with improved agreement upon CF application. Correlation between AVA and peak velocity, mean pressure gradient and velocity ratio was unchanged with the CF. CF application reclassified 2 mild cases (11%) to no AS, 13 moderate cases (33%) as mild and 21 severe cases (39%) were reclassified as moderate.

**Conclusions:** Regarding the feasibility, 1 out of 5 patients could not have LVOT area planimetry performed. This, in addition to the inferior operator variability of 3D measurements, limits the role of 3D imaging and the CF in daily practice. Further research determining the agreement of 3D measurements with the gold standard of computer tomography is required to substantiate routine CF use.

**Supporting Figures:**

**Figure 1 (abstract ABS003):**
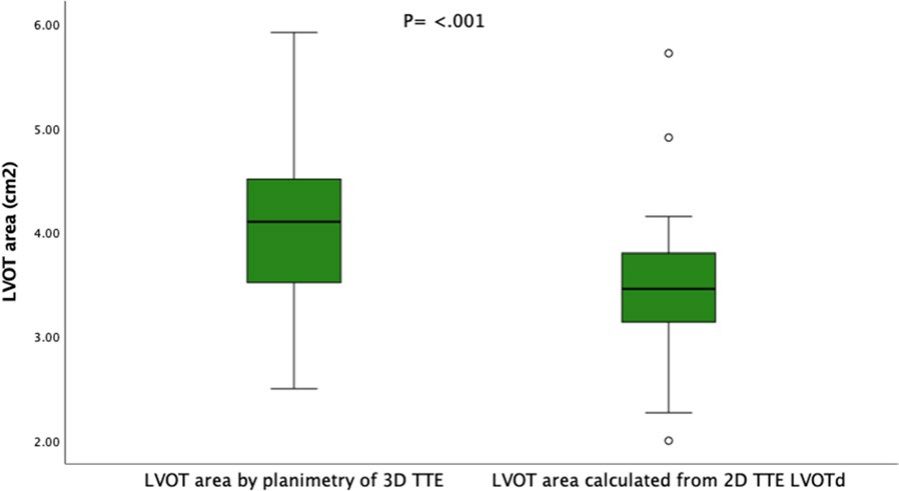
A box plot demonstrating that when calculated from the 2D LVOTd, the LVOT area is significantly underestimated compared to planimetry of the LVOT area from the 3D TTE image (P ≤ 0.001). Abbreviations = LVOT: left ventricular outflow tract, TTE: transthoracic echocardiogram, 2D: two-dimensional, 3D: three-dimensional

**Figure 2 (abstract ABS003):**
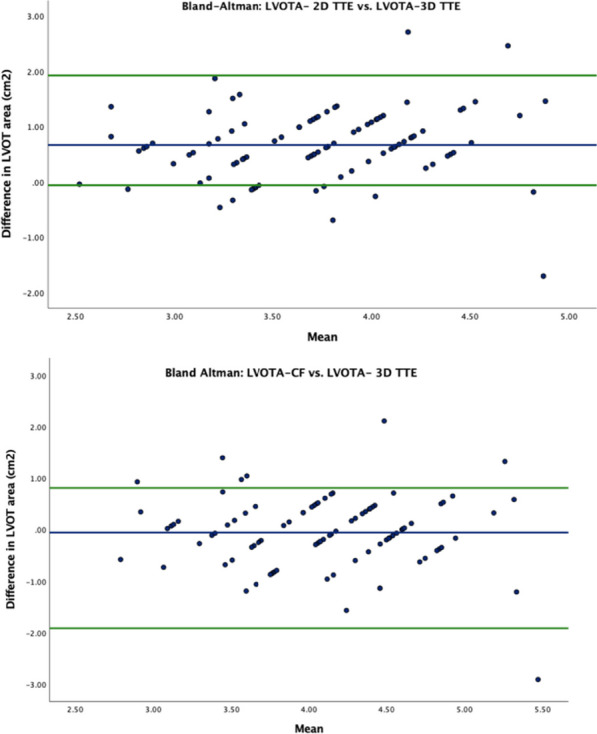
Bland-Altmann plots showing the disagreement between LVOT area measured by planimetry and calculated from 2D LVOTd (top) (mean difference, P < 0.001). CF application improved agreement between the two methods (bottom) (mean difference, P = 0.446). Differences were calculated as 3D LVOT area − LVOT area calculated from 2D LVOTd (with/without the CF). The blue line represents the mean difference, and the green lines are the 95% confidence intervals (upper and lower limits of agreement). Abbreviations = LVOTd: left ventricular outflow tract diameter, LVOTA: left ventricular outflow tract area, TTE: transthoracic echocardiogram, CF: correction dimensional, 3D: three-dimensional

**Figure 3 (abstract ABS003):**
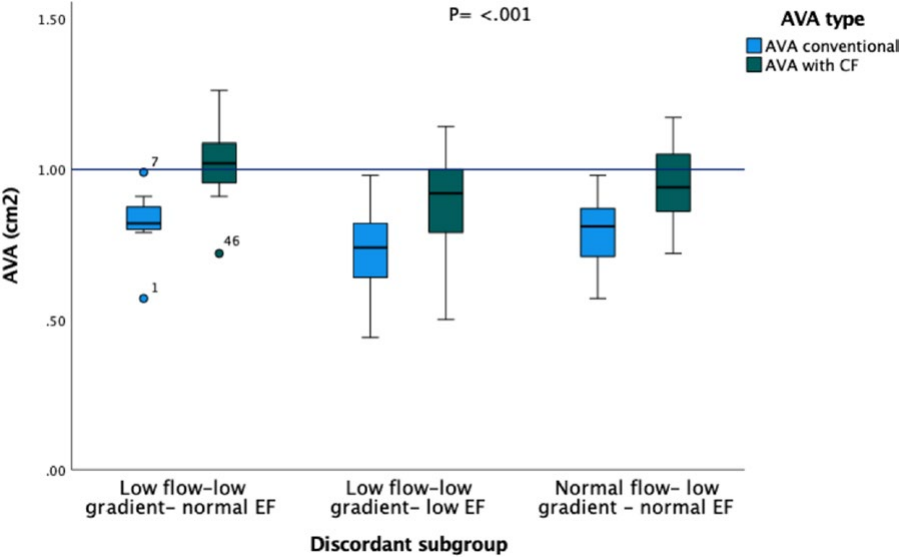
A box plot displaying 3 subgroups of discordant severe AS. Application of the CF resulted in a large proportion of patients moving to the moderate group (above the blue line). The most effected being the low flow-normal EF group where 71% were reclassified as moderate (a total interquartile range). The circles represent outliers. Abbreviations = AS: aortic stenosis, CF: correction factor, EF: ejection fraction

## ABS004 Implementing the new BSE methods and reference ranges for the Proximal Ascending Aorta and the impact on downstream testing—experience of a District General Hospital

### Julianne Doherty^1^, Chris Ellis^1^, Eveline Lee^1^

#### The Shrewsbury and Telford Hospital NHS Trust, UK

*Echo Research & Practice* 2024, **11(Suppl 1)**:ABS004

**Background:** In 2020 the BSE updated the methods and reference values for assessing the proximal ascending aorta (PAA). It is important to quantify how implementing these methods alter the rate of ‘dilated’ PAAs identified by echocardiography, and how this will impact the wider service and patient pathway.

**Purpose:** To compare the rate of dilated PAAs detected by the current BSE methods, and two other methods of assessing the PAA in our patient population.

**Methods:** All transthoracic echocardiograms where the PAA was measured between January 2018 and December 2019 were included. Studies with incomplete demographics or bicuspid aortic valves were excluded. The PAA was indexed to height (Method 1), body surface area (BSA) (Method 2) and height^2.7^ (Method 3), compared to the corresponding normal reference values and classified as ‘dilated’ or ‘non-dilated’ accordingly.

The rate of ‘dilated’ proximal ascending aortas were compared using Chi-squared test.

**Results:** 11,828 studies were identified. 2189 were removed due to incomplete patient demographics and 27 with bicuspid aortic valves. 2710 studies were removed as Method 2 does not provide reference values for patients < 45 and Method 3 > 80 years old. 6902 studies were included in the analysis.

Method 1 classified significantly more PAAs as ‘dilated’ (31%, AUC = 0.930) compared to Method 2 (10%, AUC = 0.841) and 3 (3%, AUC = 0.921) (*X*^2^(1, N = 6902) = 2435.8, p < 0.001).

**Figure 1 (abstract ABS004):**
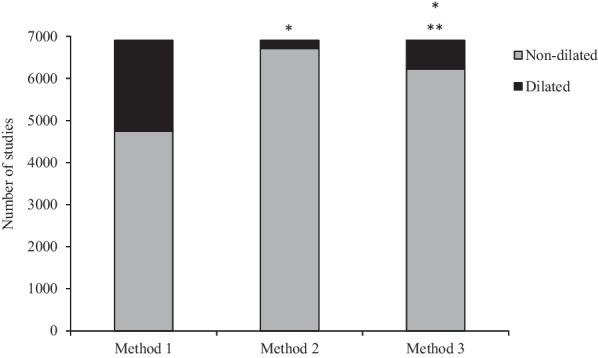
A comparison of number of Proximal Ascending Aortas classified as dilated using three different methods of normalising and assessing the proximal ascending aorta to body size; Method 1—height and sex, Method 2—body surface area, age and sex and Method 3—height^2.7^, age and sex. *Significantly different from Method 1 (p < 0.001). **Significantly different to Method 2 (p < 0.001)

**Figure 2 (abstract ABS004):**
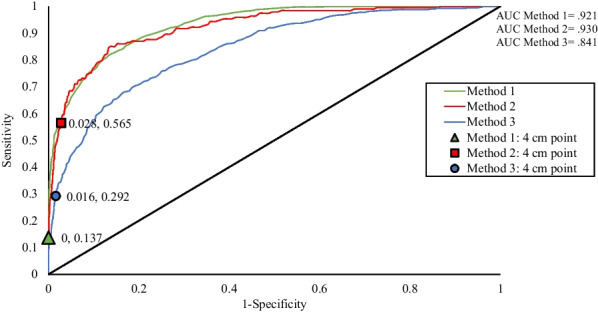
Receiver Operating Characteristic (ROC) curves of three methods for assessing the size of the proximal ascending aorta (PAA) on echocardiography; BSE recommended methods using height and sex (Method 1, green), body surface area, age and sex (Method 2, red) and height^2.7^, age and sex (Method 3, blue). Sensitivity and 1-specificty values for each method at the PAA diameter of 4 cm is plotted. Of the 6902 studies, 306 PAAs were > 4 cm. Method 1 classified all PAAs > 4 cm and 1885 < 4 cm as dilated; Method 2 classified 111 PAAs > 4 cm and 82 < 4 cm as dilated; and Method 3 classified 203 PAAs > 4 cm and 476 < 4 cm as dilated

**Conclusion:** Adopting the 2020 BSE recommended methods significantly increase the detection rate of dilated PAAs in our patient population. This will impact subsequent downstream testing, affecting resource planning and patient journey.

## ABS005 The impact of positional blood pressure variation on myocardial work in patients with severe aortic stenosis

### Peter Luke^1^, Mohammad Alkhalil^2^, Mario E. Diaz Nuila^2^, Ioakim Spyridopoulos^2,3^, Christopher Eggett^1^

#### ^1^School of Medicine, Newcastle University, Newcastle upon Tyne, UK; ^2^Cardiothoracic Centre, Freeman Hospital, Newcastle upon Tyne, UK; ^3^Institute of Translational and Clinical Research, Newcastle University, Newcastle upon Tyne, UK

*Echo Research & Practice* 2024, **11(Suppl 1)**:ABS005

**Introduction:** Myocardial work incorporates myocardial deformation imaging and non-invasive blood pressure (BP) readings. It is well established that BP differs between supine and seated positions and there are reports that inter-arm BP variability exists in some patients with severe aortic stenosis.

**Purpose:** Evaluation of body position differences in BP and consequent effect upon myocardial work is currently unknown. This study aims to assess if BP variation due to body position or inter-arm BP differences significantly alter myocardial work.

**Method:** 43 subjects with severe aortic stenosis scheduled for transcatheter aortic valve intervention were prospectively recruited. Each had an echocardiogram prior to intervention, pre-discharge and at six-week follow-up. Optimised apical four, two and long-axis views were used for measurement of global longitudinal strain. BP was recorded seated and in the left lateral decubitus position using a validated automated BP machine. BP was added to mean transvalvular aortic gradient for the calculation of myocardial work before intervention. Global work index (GWI), global constructive work (GCW), global wasted work (GWW) and global work efficiency (GWE) were compared to assess differences attributable to BP variation.

**Results:** Significant increases in GWI (1841 ± 652 mmHg% vs 1782 ± 639 mmHg%, *P* = 0.005), GCW (2305 ± 728 mmHg% vs 2236 ± 728 mmHg%, *P* = 0.015) and GWW (263 ± 131 mmHg% vs 255 ± 125 mmHg%, *P* = 0.019) were observed when using seated BP compared to left lateral decubitus recorded BP. There was no significant difference in any myocardial work parameters due to inter-arm BP variations.

**Conclusion:** BP for the purpose of myocardial work should be consistently recorded in the left lateral decubitus position to minimise the small but significant influence of positional blood pressure variation.

## ABS006 The clock is ticking: an innovative echocardiogram efficiency improvement programme at Guy’s & St Thomas’ NHS Trust vs national picture

### Dario Freitas^1,†^, Jenna Smith^1,†^, Camelia Demetrescu^1^, Nathan Proudlove^2^

#### ^1^Guy’s and St Thomas’ Hospital, NHS Trust, London, UK; ^2^The University of Manchester, UK

*Echo Research & Practice* 2024, **11(Suppl 1)**:ABS006

^†^Joint first authors.

**Background:** Echocardiography (TTE) is one of the most requested non-invasive cardiac diagnostic tests in the NHS with demand often exceeding capacity. This is reflected in the national dataset, where post-pandemic data show around 45% of TTE outpatients wait longer than the 6-week NHS England target of 1% set in 2008, compared with 7% pre-pandemic.

These national data show Guys & St Thomas’ (GSTT) performance was 30% breaching in February 2022 (Figure 1). Whilst there is no equivalent national dataset for inpatient TTE, many trusts, including GSTT, experience similar challenges despite robust triaging and the BSE’s TTE timeframe guidelines.

**Purpose:** To understand GSTT TTE performance and apply innovative quality improvement (QI) methodologies to inpatient and outpatient pathways to improve service efficiency and reduce waiting times.

**Methods:** Several change ideas were designed, tested & refined through plan-do-study-act (PDSA) cycles (Figures 2, 3).

**Results:** Around 20 outpatient appointments per week are no longer wasted (increase in activity of over 1000 TTEs per year) with a significant reduction in 6-week waiting-time breaches to below 5%.

Inpatient TTE service efficiency improved from 73 to 88% of referrals performed within 1-day or less. Levels of staffing and number of TTE referrals consistently stayed the same throughout the QI project.

**Conclusions:** Clinical Scientists leading rigorous QI initiatives can significantly improve TTE service efficiency in the post-pandemic era despite national workforce shortages and with no additional resources. Other ongoing elements of our QI programme include work on reducing DNA rates, inappropriate TTE referrals and maintaining TTE service efficiency despite major IT changes in the trust.

**Figure 1 (abstract ABS006):**
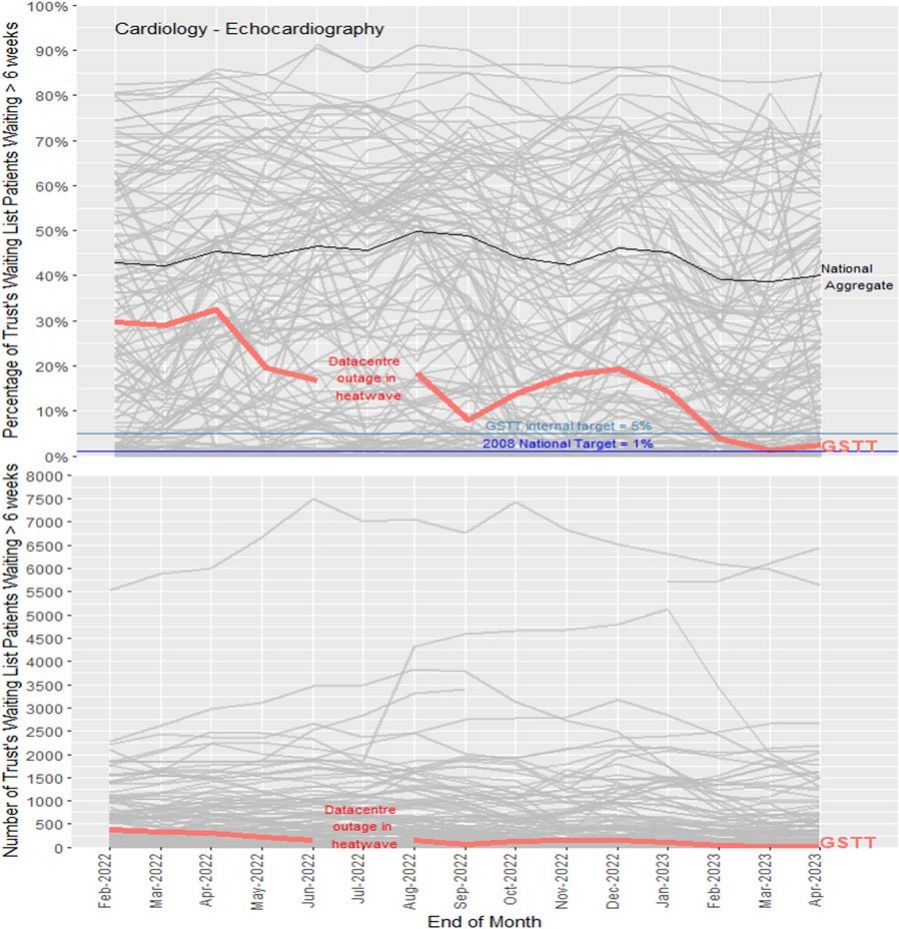
National diagnostic set data. Each line is the performance of an NHS trust (N = 134) on the 6-week-wait benchmark as a % of the Echocardiography waiting list (top) and the number of patients (bottom)

**Figure 2 (abstract ABS006):**
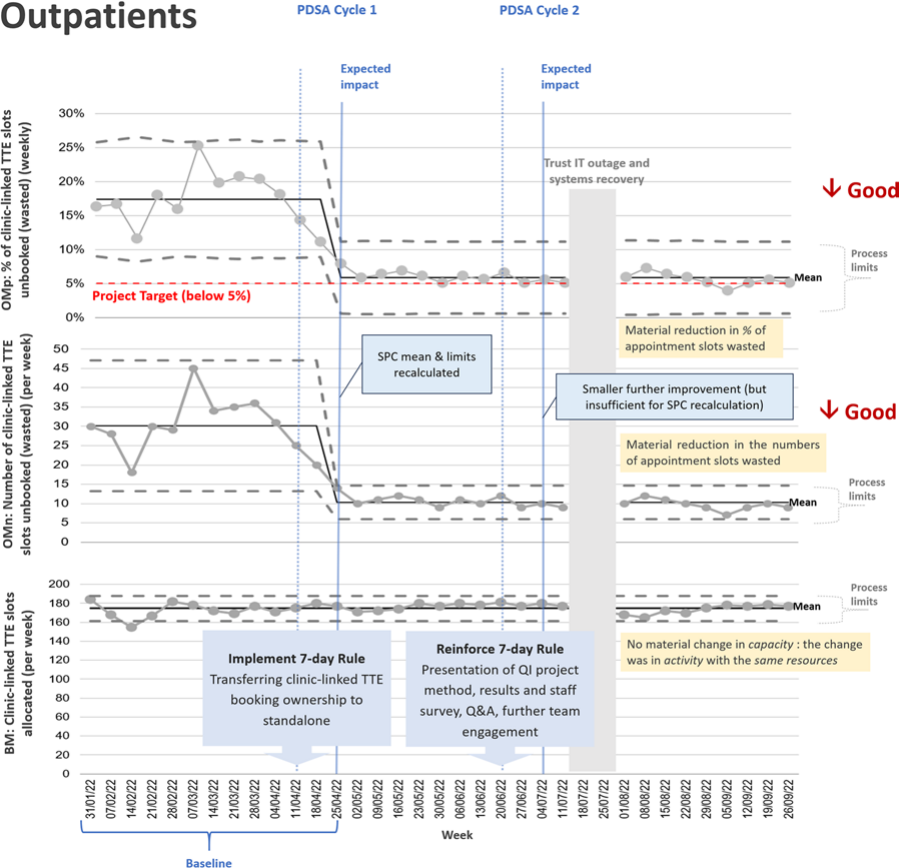
TEE outpatient QI project—SPC charts of metrics over time. OM = outcome metric; BM = balancing metric; PDSA = plan-do-study-act cycle

**Figure 3 (abstract ABS006):**
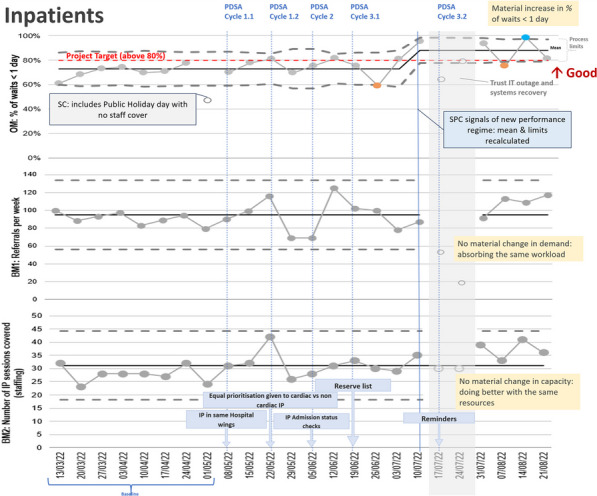
TEE inpatient QI project—SPC charts of metrics over time. OM = outcome metric; BM = balancing metric; PDSA = plan-do-study-act cycle; IP = inpatient; SC = special cause (non-random behavior with assignable cause)

## ABS008 Left atrial strain and left atrioventricular coupling index: improving the detection of cardiotoxicity

### Tom Adlington^1^, Georgina Gullick^1^, Jasmine White^1^, Ellie Jones^1^, Rebecca Bowen^1^, Dan Augustine^1^

#### ^1^Royal United Hospital Bath, UK

*Echo Research & Practice* 2024, **11(Suppl 1)**:ABS008

**Background:** Current guidelines utilize markers of systolic function (ejection fraction (EF)/global longitudinal strain (GLS)) to define cancer treatment related cardiotoxicity (CTRCT) in those receiving anti-HER2 therapy.

**Purpose:** To assess novel markers of left atrial function and LV coupling to identify CTRCT. Left atrial reservoir strain (LArS) and the left atrio-ventricular coupling index (LACI, ratio of E/e′ to GLS) were assessed in addition to EF/GLS.

**Methods:** 25 patients who developed CTRCT whilst receiving anti-HER2 therapy represent the ‘toxicity’ group. A 25 patient cohort (matched for demographics and cancer treatment) not developing CTRCT were the control group. Retrospective analysis of EF, GLS, LArS and LACI was undertaken. Probable cardiotoxicity (pCTRCT) was indicated by a drop in GLS/LArS/LACI by 15%/15%/10% respectively.

**Results:** All parameters were significantly worse in the final scan of the toxicity group compared with baseline and there was no significant change in the controls (Table 1). Time for pCTRCT was similar between GLS/LArS/LACI and better than EF CTRCT (Table 2). LArS and LACI had significantly higher EF at the point of pCTRCT compared with GLS (Figure 1).

**Table 1 (abstract ABS008):**
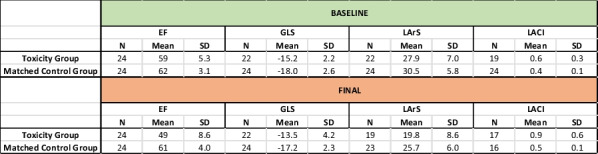
Sample statistics summary

**Table 2 (abstract ABS008):**
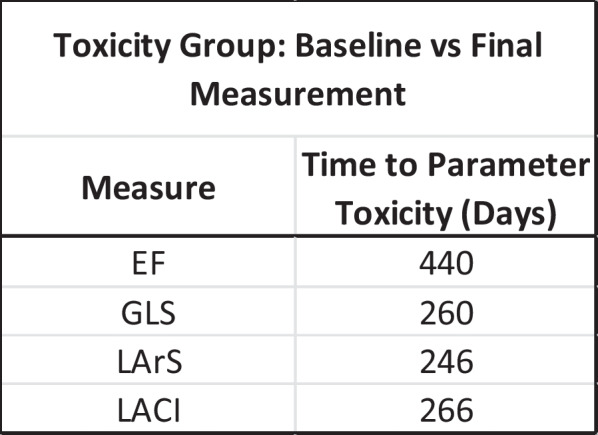
Time for parameter to indicate CTRCT

**Figure 1 (abstract ABS008):**
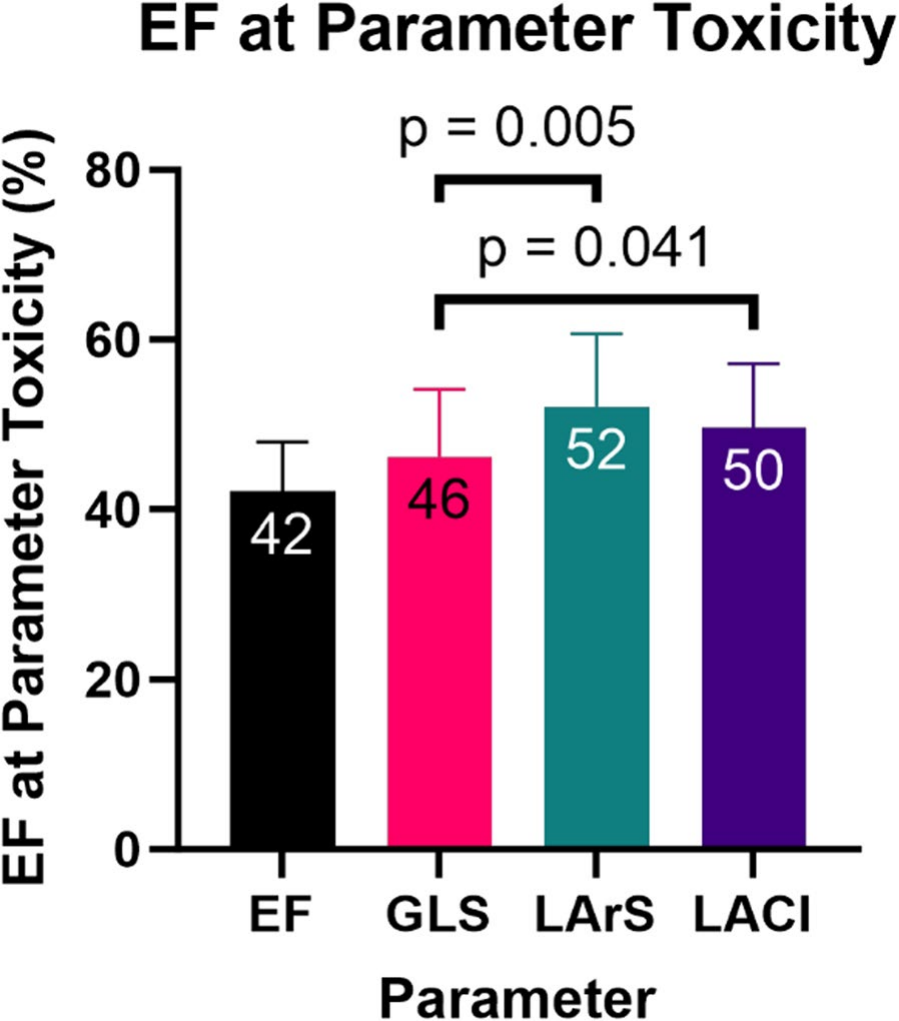
EF at scan where parameter indicates cardiotoxicity. EF criteria: absolute value drop by > 10% to value below 50%. GLS criteria: relative drop of > 15%. LArS criteria: relative drop of > 15%. LACI criteria: Relative increase of > 10%. T-Test significance analysis shown

**Conclusion:** Novel measures of left atrial function and left atrioventricular coupling can diagnose pCTRCT at significantly higher EF than GLS. Left atrial function and left atrioventricular coupling are possible markers of pCTRCT, potentially enabling alterations in cardioprotective and cancer management at an earlier stage than if EF was used alone. Prospective studies utilising LArS and LACI are needed.

## ABS009 Optimising the usage of abbreviated cardiac ultrasound scanning in specific cohort of outpatient referrals

### Sita Thapa Magar^1^, Delfin Encarnacion^2^, James Malcolmson^2^, Michelle Navalesh^3^, Guy Lloyd^2^, Sanjeev Bhattacharyya^2^

#### ^1^St Helier and Epsom University Hospital, NHS Foundation Trust, London, UK; ^2^Bart’s Health NHS Trust, London, UK; ^3^St George’s University Hospitals NHS Foundation Trust, London, UK

*Echo Research & Practice* 2024, **11(Suppl 1)**:ABS009

**Background:** The project is based on a clinical audit utilising an Abbreviated or targeted scanning protocol (AEP) for specific cardiac pathologies. Echo referrals were triaged for eligibility and feasibility for the AEP, such as patients referred from murmur detection, arrhythmia presentation or rapid access chest pain clinic. Scans were conducted by a Senior Cardiac Physiologist with years of competency and quality assurance feedback.

**Purpose:** The audit investigated the role of an AEP in improving access to echocardiography services and clinical outcomes of specific outpatient referrals.

**Methods:** A total retrospective sample of 373 lists were included for an AEP scan. The referrals were specifically for outpatient and were triaged after meeting the eligible criteria. The Echo scans were performed by a level-2 senior sonographer with capability to assess from limited images taken and decide on adding imaging parameters such as spectral Doppler if required.

**Results:** Most referrals were specific for ventricular function assessment, structural abnormalities, and murmur detection. Arrhythmia indication consists of 38% (n = 165), systolic function assessment 21% (n = 80), and murmur 20% (n = 75) of cases. 55% of the referrals were successfully performed using AEP versus 45% requiring spectral Doppler or full study. 66% of the abbreviated scans were normal findings, 11% detected regional-wall-motion or systolic function abnormalities, and 23% detected minor valvular dysfunction. The median scanning time for abbreviated scanning was 11 min (IQR 10–12 min).

**Conclusions:** Our internal clinical service audit study demonstrates that an abbreviated echocardiography protocol can improve access to echocardiography services by increasing scheduling capacity without compromising diagnostic performance in a low-risk outpatient population.

## ABS010 Evaluating adherence to aortic stenosis guidelines in a valvular heart disease surveillance clinic—a 5-year audit

### Zak Rossaye^1,2^, Cristina Constantin^1^, Sarah Salih^3^

#### ^1^Princess of Wales Hospital, Bridgend, UK; ^2^Manchester Metropolitan University, UK; ^3^Keele University School of Medicine, UK

*Echo Research & Practice* 2024, **11(Suppl 1)**:ABS010

**Background:** Aortic stenosis (AS) is the most common native heart valve disease. Guidelines recommend monitoring patients with severe AS every 6-months, ideally in a dedicated valvular heart disease (VHD) clinic. The COVID-19 pandemic caused severe healthcare disruptions and affected patients’ access to the entire aortic stenosis pathway, from surveillance to intervention.

**Purpose:** This audit aimed to evaluate the current compliance of our VHD clinic with European guidelines for the surveillance and management of patients with severe AS. We aimed to determine whether patients were followed up within the guideline-accepted timeframe, whether they received prompt intervention, and assess the impact of COVID-19 on patient’s access to the service.

**Methods:** This retrospective, single-centre, observational study identified patients with severe AS assessed in the VHD clinic at the Princess of Wales Hospital between 2017 and 2022. Data collected included appointment dates, time intervals between appointments, referral date for intervention, date and type of intervention, patient status, and date of death. We also compared surveillance and intervention data before and during the COVID-19 pandemic.

**Results:** The average time between specialist visits for patients with severe aortic stenosis was 9.7 ± 6.8 months (Figure 1). During the COVID-19 pandemic, the mean follow-up time increased significantly from 6.77 ± 3.77 to 13.52 ± 7.79 months (*p* < *0.001*) (Figure 2). No significant difference in survival was found between patients seen within six months or after six months (*p* = *0.743*). Out of the cohort, 49% of patients (200) were referred for intervention, and the mean waiting time for intervention was 4.95 months ± 4.25 months (range: < 1 to 30.53 months). The mean time frame from referral to intervention decreased from 5.89 ± 4.95 months to 3.73 ± 2.76 months since the start of the pandemic (*p* = *0.002*) (Figure 3). Out of the patients who received intervention, 49.02% (75) had SAVR, 49.02% (75) had transcatheter aortic valve implantation (TAVI), and 1.96% (3) had BAV. Of the whole cohort, 54.7% (223) were still alive at the end of the audit period while 45.3% (185) had died (Table 1).

**Conclusions:** This cohort of patients has a very high mortality rate due to severe AS and significant comorbidities in old age and so timing of intervention is crucial for their outcome. This audit found that follow-up of patients with severe AS within the recommended six-month timeframe was challenging, particularly during the COVID-19 pandemic. However, patients who were referred for intervention had shorter referral-to-intervention times during the pandemic. We suspect this is due to the improved access to TAVI, reduced inpatient stay and potential exposure of patients to the COVID-19 infection and faster recovery times.

**Figure 1 (abstract ABS010):**
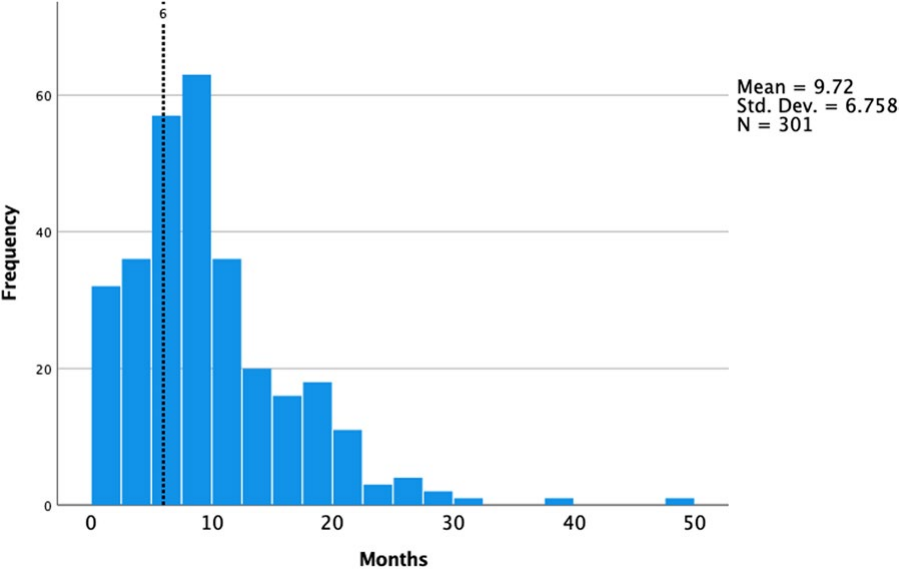
Average follow up time of patients with severe aortic stenosis. Dashed line represents the guideline recommended follow up time (6 months)

**Figure 2 (abstract ABS010):**
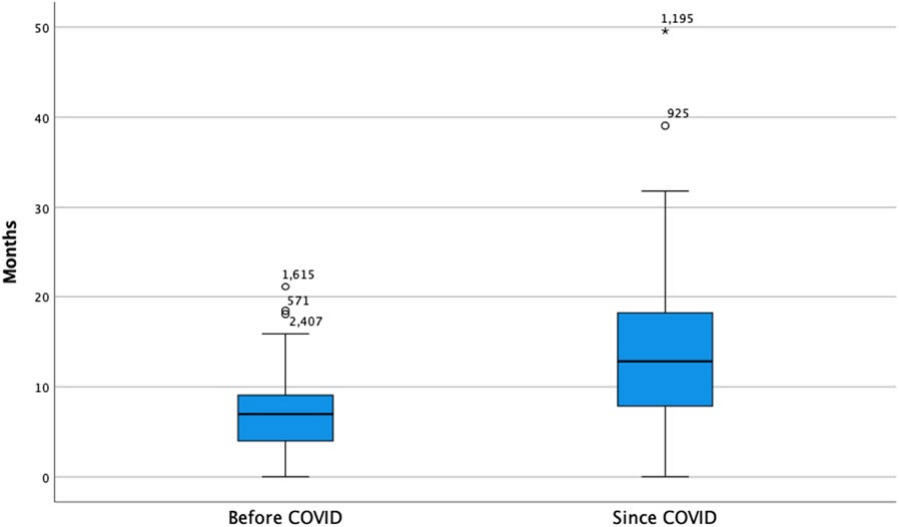
Boxplots demonstrating the significant difference in follow-up time before and after the COVID19 pandemic

**Figure 3 (abstract ABS010):**
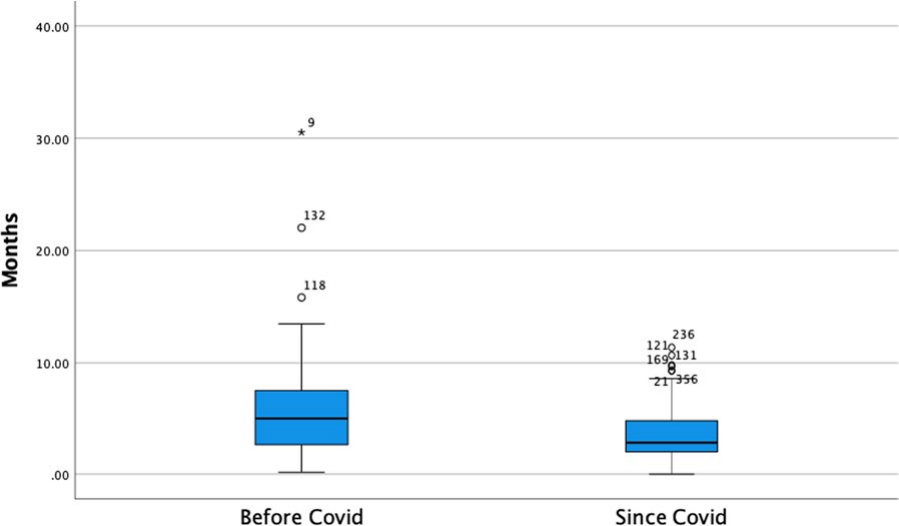
Boxplots demonstrating the significant difference in referral-to-intervention time before and after the COVID19 pandemic

**Table 1 (abstract ABS010):** Patient’s age, mortality, echocardiographic and NT-proBNP data

	Male (n = 216)	Female (n = 192)	*p*-value
	Mean ± SD	Mean ± SD	
Age (years)	82.81 ± 8.88	84.08 ± 8.85	*0.15*
Mortality (% alive/% dead)	53%/47%	57%/43%	*0.419*
Aortic valve area by planimetry (cm^2^)	0.81 ± 0.25	0.73 ± 0.24	< *0.001*
Aortic valve area by continuity (cm^2^)	0.80 ± 0.24	0.72 ± 0.22	< *0.001*
Aortic valve peak velocity (m/s)	4 ± 0.77	4.04 ± 0.8	*0.667*
Aortic valve peak gradient (mmHg)	66.46 ± 23.88	68.01 ± 24.54	*0.604*
Aortic valve mean gradient (mmHg)	40.06 ± 15.27	41.86 ± 16.38	*0.257*
NT-proBNP (pg/ml)	5026.33 ± 8501.21	5032.25 ± 9484.95	*0.429*

## ABS012 Inter-observer variability in the assessment of left ventricular size and systolic function

### Yande Kasolo^1^, James Redfern^2^

#### ^1^Royal Liverpool University Hospital, UK; ^2^Countess of Chester Hospital, UK

*Echo Research & Practice* 2024, **11(Suppl 1)**:ABS012

**Background:** Echocardiography remains the workhorse of cardiac imaging, and reproducibility of results within departments and across sites is key to consistent decision-making for patients. The Intraclass Correlation Coefficient (ICC) allows objective assessment of the variation in reporting of the same study by sonographers both within the same department and also across different locations. An ICC of > 0.75 represents excellent correlation.

**Purpose:** To audit the Inter-observer variability (IOV) of left ventricular size and functional assessment in and between two hospital Trusts.

**Method:** Sonographers and physicians across two Cardiorespiratory Departments reported independently on the IVSd, LVIDd, PWd, EF by Simpson’s and visually estimated EF of the same 5 anonymised studies. The Two-factor Anova Test, and ICC were calculated for each parameter, and the results reported.

**Results:** Data were collected from 20 participants (Trust 1 n = 11, Trust 2 n = 9) and the ICC for each parameter recorded (Table 1). Excellent correlation was seen at both sites for assessment of EF with fair (Trust 1) and poor (Trust 2) correlation for the PWd measurement. The assessment of EF was comparable between both trusts (Figures 1, 2).

**Conclusion:** The results highlight the inherent variability in echocardiography reporting and demonstrate a method by which this variability can be assessed objectively and be audited. By understanding the ICC within a department, and between departments across the NHS, clinicians can feel confident that reported results are reproducible, irrespective of the performing sonographer. This should form an aspect of QA and clinical governance that ensures consistent access to guideline-directed therapy.

**Table 1 (abstract ABS012):**
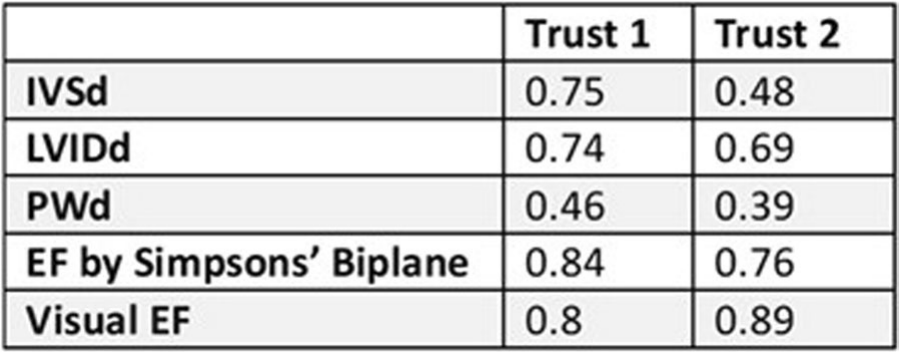
Intraclass Correlation Coefficient values for 2D echo measurements

**Figure 1 (abstract ABS012):**
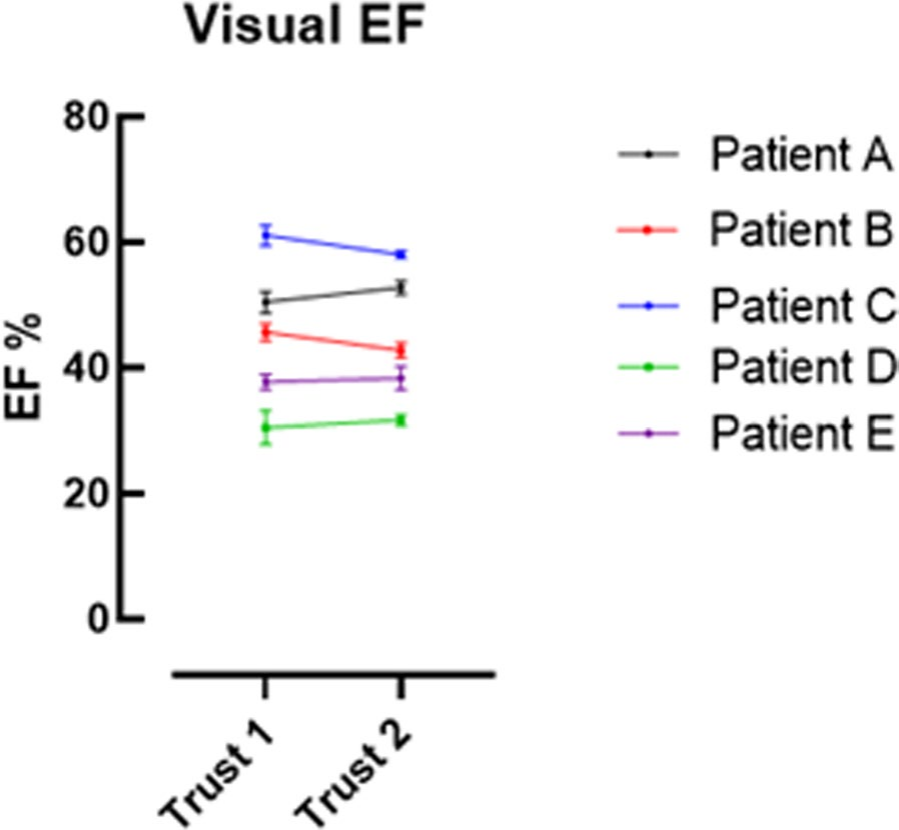
Graph demonstrating standard error of the mean for the visual assessment of Left Ventricular Ejection Fraction

**Figure 2 (abstract ABS012):**
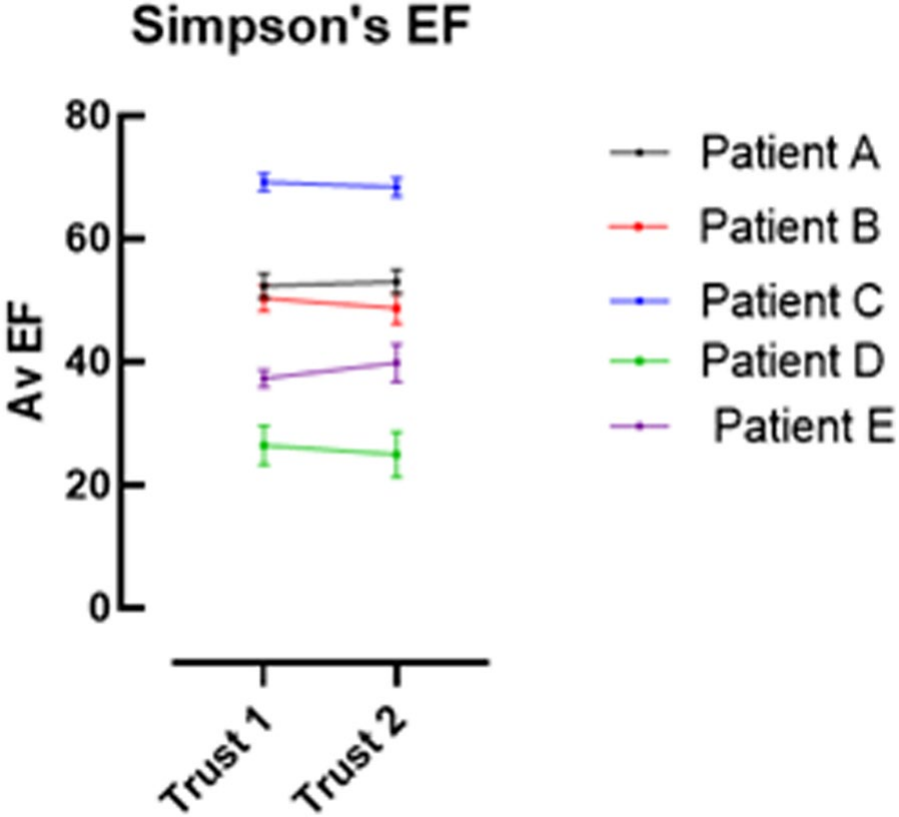
Graph demonstrating standard error of the mean for the assessment of LV systolic function by Simpsons’s Biplane method

## ABS013 Patient satisfaction: a single-centre study assessing the impact of modifiable and non-modifiable determinants

### Kai Li Chew^1^, James Willis^2^, Freya Henry^2^, Sarah Trippier^2^, Claire Lowe^2^, Rhiannon Edwards^1^, Daniel Augustine^2^

#### ^1^University of Bath, UK; ^2^Royal United Hospitals Bath, UK

*Echo Research & Practice* 2024, **11(Suppl 1)**:ABS013

**Background:** Patient satisfaction is a critical indicator of the quality of healthcare services. The National Health Service face challenges with waiting times and backlogs. Echocardiography plays a pivotal role in the management of cardiac conditions. Understanding patient experience is essential in improving patient-centred care. This cross-sectional study assessed the degree of patient satisfaction and its relationship with modifiable and non-modifiable factors.

**Method:** Patients (n = 54) attending a busy district general hospital for their first echocardiogram were recruited. They were asked to complete a survey which included variables relevant to patient satisfaction (e.g., demographics, self-perceived health, discomfort during the echocardiogram, expectation regarding healthcare services, physical environment, clinical competence, accessibility and waiting time). Descriptive statistics and linear regression analysis was performed to assess the associations between these factors and overall patient satisfaction.

**Results:** Patients reported high satisfaction for the following: overall experience of having an echocardiogram; sonographers interpersonal care/communication, information provision, clinical competence; accessibility and waiting time on the day. Moderate satisfactions were reported for the physical environment of the hospital. Two modifiable factors and two non-modifiable factors were found to be associated with overall satisfaction towards echocardiography. These were interpersonal communication, information provision, self-perceived health and discomfort.

**Conclusion:** Overall, generally patients were satisfied with their experience. Understanding modifiable and non-modifiable factors for individual centres may help to enhance patients’ experience and healthcare quality.

## ABS014 Bridging the gap: focused echo clinics in a resource-limited system

### Dan Mort^1^, Serina Sidhu^1^, Jo Wynn^1^, Amy Ewen^1^, Amanda Searle^1^, Katrin Balkhausen^1^

#### ^1^Royal Berkshire Hospital, Reading, UK

*Echo Research & Practice* 2024, **11(Suppl 1)**:ABS014

**Background:** In early 2022, staff shortages and post-pandemic pressures in our large DGH led to a backlog of over 1500 outpatient echocardiograms, with an average wait of nearly 6 months. This posed a substantial risk of late or missed diagnosis to our patients.

**Purpose:** A “Focused Echo Clinic” (FEC) model was proposed to facilitate higher scan turnover, timely diagnosis and triage to further management.

**Methods:** A pilot period of 4 days of two parallel FECs with 15 min per scan was executed in April 2022, with immediate audit and internal quality assurance. Pre-defined patient selection criteria and a standard operating procedure (SOP) were developed. All scans were performed by senior Band 7 echocardiographers. Patients not needing follow-up were audited at 9 months for adverse outcomes. FECs between May and October 2022 were also audited.

**Results:** 673 focussed scans on 29 days over a 6-month period were performed. 74% needed no further follow-up. A substantial burden of varied functional and structural pathology was diagnosed and triaged to further imaging and clinics (Figs. 1, 2). All 161 pilot scans were SOP-compliant, with 81% exceeding that minimum standard. There were no adverse outcomes at 9 months that a full scan would have prevented (Fig. 3). FECs halved the mean waiting time to 3 months. Patient feedback was excellent, with no complaints from GPs or hospital colleagues.

**Conclusion:** A FEC model can effectively facilitate timely diagnoses and management of serious cardiac pathology with good medium-term safety. This proof-of-concept may help resource-limited departments to address rising demand whilst keeping patient safety central.

**Figure 1 (abstract ABS014):**
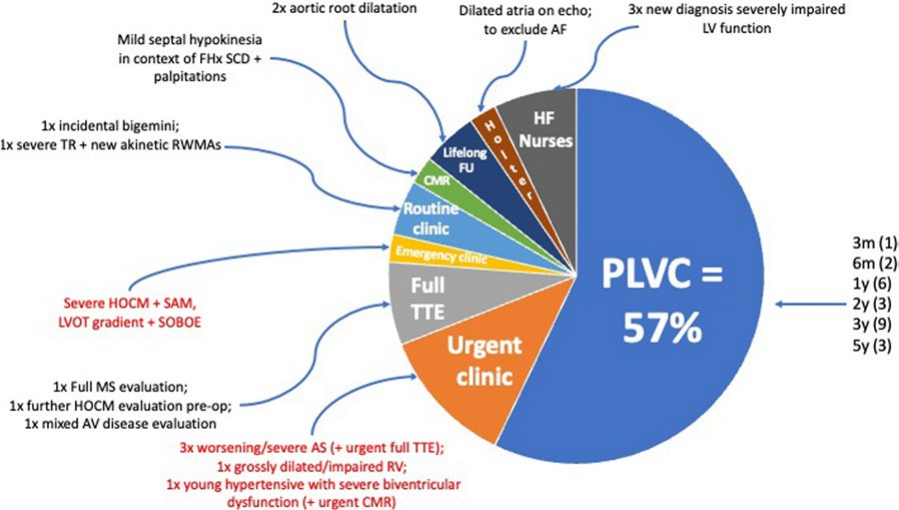
April 2022 Pilot Findings and Triage Destinations of patients requiring follow-up after focused echo. PLVC = physiologist-led valve clinic (recommended time-to-clinic displayed to right). SCD = sudden cardiac death

**Figure 2 (abstract ABS014):**
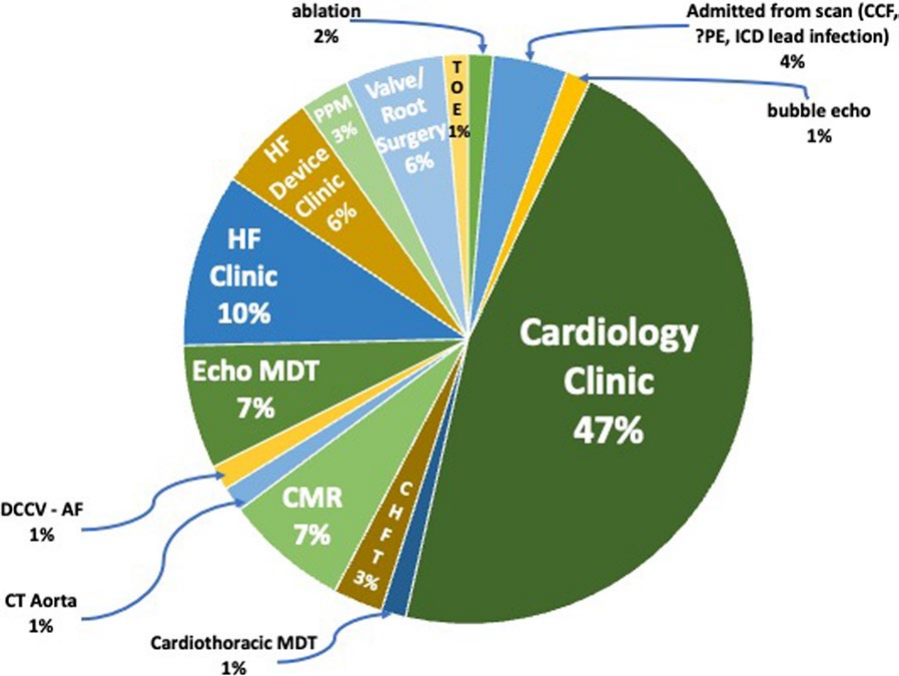
May–October 2022 Breakdown of 71 patients (14%) requiring follow-up other than a routine formal echo. CHFT = community heart function team

**Figure 3 (abstract ABS014):**
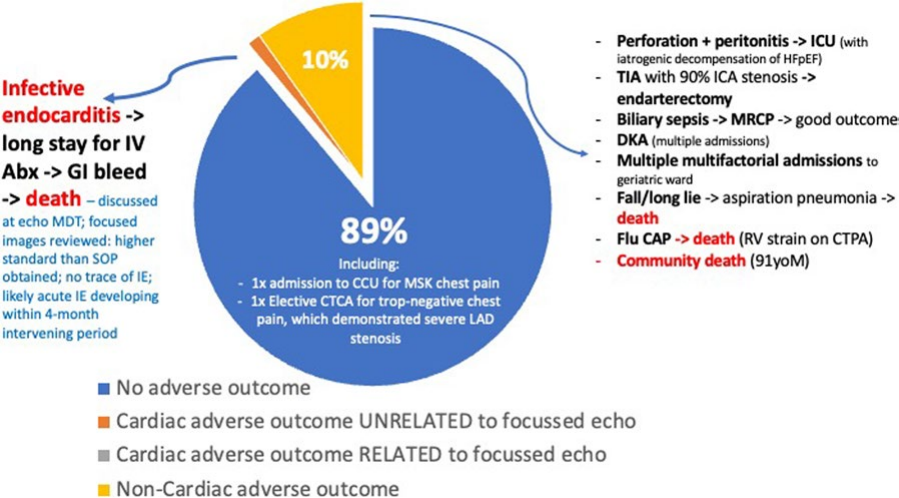
9-month safety audit of April 2022 pilot patients requiring no specific follow-up after focused scan

## ABS015 Temporal changes in stress echocardiography practice across the UK: insight from the BSE-NSTEP multi-centre study

### Casey Johnson^1^, Samuel Krasner^1^, Emma Mao^1^, William Woodward^1^, Annabelle McCourt^1^, Cameron Dockerill^1^, Mark Monaghan^2^, Roxy Senior^3^, Daniel Augustine^4^, Maria Paton^5^, Jamie O’Driscoll^6^, David Oxborough^7^, Keith Pierce^8^, Shaun Robinson^9^, James Willis^4^, Paul Leeson^1^, on behalf of the EVAREST/BSE-NSTEP Investigators

#### ^1^Cardiovascular Clinical Research Facility, University of Oxford, UK; ^2^Kings College Hospital NHS Foundation Trust, London, UK; ^3^Northwick Park Hospital-Royal Brompton Hospital, London, UK; ^4^Royal United Hospitals Bath NHS Foundation Trust, UK; ^5^University of Leeds and Leeds Teaching Hospitals NHS Foundation Trust, UK; ^6^Canterbury Christ Church University, UK; ^7^Liverpool Centre for Cardiovascular Science, UK; ^8^Manchester University NHS Foundation Trust, UK; ^9^Imperial College Healthcare NHS Trust, London, UK

*Echo Research & Practice* 2024, **11(Suppl 1)**:ABS015

**Background:** Stress echocardiography (SE) is widely used to detect coronary artery disease (CAD), but recent changes in clinical guidelines, clinical practice, and public health may have shifted both the population being referred for SE and the manner in which SE is conducted.

**Purpose:** To identify temporal changes in the patient demographics of those being investigated with SE and the method of SE practice across NHS Trusts in the UK.

**Methods:** Participant demographics and SE procedure details were collected for 13,781 participants as part of the multi-centre BSE-NSTEP study. Data was acquired from March 2015–September 2020 as part of Groups 1 and 2 of the study, and October 2020–May 2023 as Group 3. Comparisons were conducted for the overall cohorts and further according SE result (positive or negative). Descriptive statistics were investigated as frequencies and medians [interquartile range (IQR)]. Comparison of discrete data was conducted using Pearson’s χ^2^ tests.

**Results:** Data was collected for 7504 participants across 30 NHS Trusts as part of Group 1/2 and a further 6,277 across 34 NHS Trusts as part of Group 3. In overall cohort comparison of Group 1/2 vs Group 3 (Table 1), number of non-smokers (49.5% vs 53.6%), prevalence of hypertension (48.4% vs 52.9%), hypercholesterolaemia (39.9% vs 47.4%), diabetes (19.9% vs 22.7%), and family history of premature CAD (6.8% vs 33.7%) was higher in Group 3, while previous percutaneous coronary intervention was lower (32.5% vs 21.0%) (all p < 0.001). Exercise SE (30.5% vs 37.4%), and use of contrast enhancement (71.9% vs 83.6%) was more common in Group 3, but use of Atropine in Dobutamine SE was lower (49.4% vs 44.3%) (all p < 0.001). These results are further broken into positive and negative SE result in Tables 2 and 3 respectively.

**Conclusion:** This study provides evidence of potential effects from recent clinical guideline changes and subsequent clinical practice as well as the changing landscape of patients being referred for SE.

**Table 1 (abstract ABS015):** Overall cohort comparison between Groups 1/2 and Group 3

	Group 1/2 (N = 7504)	Group 3 (N = 6277)	P-value
*Participant demographics*			
Male (%)	4236/7504 (56.4)	3620/6277 (57.0)	0.15
Median age (years) (IQR)	66 (57–73)	65 (56–73)	
Median BMI (kg/m^2^) (IQR)	28.2 (24.98–31.83)	28.27 (25.10–31.84)	
Current smoker (%)	884/7206 (12.3)	755/6065 (12.4)	0.75
Ex-smoker (%)	2755/7206 (38.2)	2062/6065 (34.0)	< 0.001
Non-smoker (%)	3567/7206 (49.5)	3248/6065 (53.6)	< 0.001
Hypertension (%)	3434/7099 (48.4)	3312/6261 (52.9)	< 0.001
Hypercholesterolaemia (%)	2836/7099 (39.9)	2970/6261 (47.4)	< 0.001
Diabetes mellitus (%)	1412/7014 (19.9)	1422/6261 (22.7)	< 0.001
Peripheral vascular disease (%)	206/7099 (2.9)	97/6258 (1.6)	< 0.001
Family history of premature CAD (%)	480/7099 (6.8)	2112/6261 (33.7)	< 0.001
Previous MI (%)	1273/7088 (18.0)	1108/6258 (17.7)	0.70
Previous PCI (%)	2311/7102 (32.5)	1312/6258 (21.0)	< 0.001
Precious CABG (%)	542/7115 (7.6)	439/6258 (7.0)	0.18
Resting RWMA (%)	1097/7452 (14.7)	718/4984 (14.4)	0.63
*Stress echocardiogram details*			
Exercise (%)	2289/7504 (30.5)	2292/6131 (37.4)	< 0.001
Pacemaker (%)	26/7504 (0.3)	31/6131 (0.5)	0.15
Dobutamine (%)	5232/7504 (69.7)	3808/6131 (62.1)	< 0.001
Atropine use in DSE (%)	2585/5232 (49.4)	1686/3808 (44.3)	< 0.001
Contrast used	5376/7480 (71.9)	5168/6180 (83.6)	< 0.001
SonoVue (%)	4957/5376 (92.2)	4158/5168 (80.5)	< 0.001
Luminity (%)	401/5376 (7.5)	1006/5168 (19.5)	< 0.001
Other (e.g. Optison) (%)	18/5376 (0.3)	4/5168 (0.1)	< 0.001
No contrast (%)	2104/7480 (28.1)	1012/6180 (16.4)	< 0.001

Patient demographics and SE procedure details for participants recruited to Group 1/2 or Group 3. Presented as n/total n. Percentages quoted in brackets

**Table 2 (abstract ABS015):** Cohort comparison for a positive stress echocardiogram result

Positive stress echo	Group 1/2 (N = 1347)	Group 3 (N = 1139)	P-value
*Participant demographics*			
Male (%)	876/1347 (65.0)	775/1139 (68.0)	0.11
Median age (years) (IQR)	68 (60–74)	67 (59–74)	
Median BMI (kg/m^2^) (IQR)	28.69 (25.6–32.2)	28.08 (25.1–31.88)	
Current smoker (%)	172/1299 (13.2)	155/1089 (14.2)	0.48
Ex-smoker (%)	520/1299 (40.0)	391/1089 (35.9)	< 0.05
Non-smoker (%)	607/1299 (46.7)	543/1089 (49.9)	0.13
Hypertension (%)	721/1347 (53.5)	681/1134 (60.1)	< 0.01
Hypercholesterolaemia (%)	668/1347 (49.6)	619/1134 (54.6)	< 0.05
Diabetes mellitus (%)	319/1347 (23.7)	344/1134 (30.3)	< 0.001
Peripheral vascular disease (%)	57/1347 (4.2)	24/1134 (2.1)	< 0.001
Family history of premature CAD (%)	65/1347 (4.8)	409/1134 (36.1)	< 0.001
Previous MI (%)	348/1322 (26.3)	259/1134 (22.8)	< 0.05
Previous PCI (%)	568/1324 (42.9)	301/1134 (26.5)	< 0.001
Precious CABG (%)	195/1326 (14.7)	133/1134 (11.7)	< 0.05
Resting RWMA (%)	483/1344 (35.9)	253/812 (31.2)	< 0.05
*Stress echocardiogram details*			
Exercise (%)	354/1347 (26.3)	309/1117 (27.7)	0.44
Pacemaker (%)	10/1347 (0.7)	13/1117 (1.2)	0.28
Dobutamine (%)	990/1347 (73.5)	795/1117 (71.2)	0.20
Atropine use in DSE (%)	530/990 (53.5)	386/795 (48.6)	< 0.05
Contrast used	971/1343 (72.3)	1026/1127 (91.0)	< 0.001
SonoVue (%)	904/971 (93.1)	903/1026 (88.0)	< 0.001
Luminity (%)	63/971 (6.5)	122/1026 (11.9)	< 0.001
Other (e.g. Optison) (%)	4/971 (0.4)	1/1026 (0.1)	0.16
No contrast (%)	372/1343 (27.7)	101/1127 (9.0)	< 0.001

Patient demographics and SE procedure details for participants recruited to Group 1/2 or Group 3 who received a positive stress echocardiogram result. Presented as n/total n. Percentages quoted in brackets

**Table 3 (abstract ABS015):** Cohort comparison for a negative stress echocardiogram result

Negative stress echo	Group 1/2 (N = 5586)	Group 3 (N = 4853)	P-value
*Participant demographics*			
Male (%)	3020/5586 (54.1)	2701/4853 (55.7)	0.10
Median age (years) (IQR)	65 (56–73)	65 (56–73)	
Median BMI (kg/m^2^) (IQR)	28.2 (24.95–31.83)	28.2 (25.07–31.71)	
Current smoker (%)	644/5393 (11.9)	566/4697 (12.1)	0.87
Ex-smoker (%)	2018/5393 (37.4)	1555/4697 (33.1)	< 0.001
Non-smoker (%)	2731/5393 (50.6)	2576/4697 (54.8)	< 0.001
Hypertension (%)	2626/5586 (47.0)	2488/4843 (51.4)	< 0.001
Hypercholesterolaemia (%)	2100/5586 (37.6)	2220/4843 (45.8)	< 0.001
Diabetes mellitus (%)	1054/5586 (18.9)	994/4843 (20.5)	< 0.05
Peripheral vascular disease (%)	147/5586 (2.6)	70/4841 (1.4)	< 0.001
Family history of premature CAD (%)	399/5586 (7.1)	1605/4843 (33.1)	< 0.001
Previous MI (%)	835/5493 (15.2)	791/4841 (16.3)	0.11
Previous PCI (%)	1594/5505 (29.0)	945/4841 (19.5)	< 0.001
Precious CABG (%)	322/5516 (5.8)	285/4841 (5.9)	0.91
Resting RWMA (%)	537/5575 (9.6)	417/3979 (10.5)	0.17
*Stress echocardiogram details*			
Exercise (%)	1641/5586 (29.4)	1919/4735 (40.5)	< 0.001
Pacemaker (%)	11/5586 (0.2)	18/4735 (0.4)	0.08
Dobutamine (%)	3960/5586 (70.9)	2798/4735 (59.1)	< 0.001
Atropine use in DSE (%)	1949/3960 (49.2)	1224/2798 (43.7)	< 0.001
Contrast used	4007/5569 (72.0)	3938/4805 (82.0)	< 0.001
SonoVue (%)	3667/4007 (91.5)	3071/3938 (78.0)	< 0.001
Luminity (%)	328/4007 (8.2)	864/3938 (21.9)	< 0.001
Other (e.g. Optison) (%)	12/4007 (0.3)	3/3938 (0.1)	< 0.05
No contrast (%)	1562/5569 (28.0)	867/4805 (18)	< 0.001

Patient demographics and SE procedure details for participants recruited to Group 1/2 or Group 3 who received a negative stress echocardiogram result. Presented as n/total n. Percentages quoted in brackets

## ABS016 Evaluation of the left ventricular function in patients with arterial hypertension and normal ejection fraction, using global longitudinal strain; a relation to left atrial function

### Rahaf M. Alshehri^1^, Petros Nihoyannopoulos^1^

#### ^1^Imperial College London (National Heart and Lung Institute), UK

*Echo Research & Practice* 2024, **11(Suppl 1)**:ABS016

**Background:** Systemic arterial hypertension is an established risk factor, the purpose of the study is to investigate whether GLS for hypertensive patients detect subclinical dysfunction before cardiac impairment is established, despite normal EF. In addition, assess the atrioventricular interdependence in hypertensive patients.

**Method:** Patients enrolled in this study who met the criteria of normal LVEF > 50% and no evidence of cardiovascular diseases, hypertensive patients (n = 86) and a healthy control group (n = 10) mean age (57 ± 15.6 years, p = 000.1). LV function was assessed using speckle tracking.

**Results:** GLS LV function was reduced in 27 (31.4%), (− 14.6 ± 2.3, p < 0.001) with normal LVEF. And were observed more often in 12 (44.4%) of the older patient population (> 60 years old) with risk factors including HTN and diabetes. LAVI was found higher among HTN patients compared to the control group (30.02 ± 12.05 ml/m^2^ vs. 22.2 ± 6.8 ml/m^2^, p = 0.007), and grew higher in HTN patients with reduced GLS (32.2 ± 12.2 ml/m^2^, p = 0.051). LA enlargement associated with reduced GLS was found in 15 (17.4%) patients. Diastolic function abnormalities were found in 56 (65.1%) patients with mean GLS values of (− 19.9 ± 4.69, p = 0.467), and reduced GLS were found in 20 patients (23.2%, p = 0.238). LVH was found in 52 (60.4%) patients, the lower GLS values were associated with concentric hypertrophy in 9 (33.3%, p = 0.002) patients.

**Conclusion:** GLS was reduced in hypertensive patients despite their normal EF, showing an association with LA enlargement.

## ABS017 Regional left ventricular longitudinal strain in resistance trained individuals using anabolic–androgenic steroids

### Harry Carpenter^1^, Florence Place^1^, Barbara N. Morrison^2^, Neil Chester^1^, Robert Cooper^1^, Ben N. Stansfield^3^, Keith P. George^1^, David Oxborough^1^

#### ^1^Research Institute of Sports and Exercise Science, Liverpool John Moores University, Liverpool, UK; ^2^School of Human Kinetics, Trinity Western University, Langley, BC, Canada; ^3^Department of Pharmacology and Toxicology, College of Pharmacy, University of Arizona, Arizona, USA

*Echo Research & Practice* 2024, **11(Suppl 1)**:ABS017

**Abstract**

**Background:** Anabolic–Androgenic Steroids (AAS) are used frequently in individuals engaged in resistance training (RT). Previous echocardiographic studies have demonstrated increased left ventricular mass (LVM) and reduced global longitudinal strain (GLS) in AAS users compared to non-users. There are, however, limited data investigating regional function in these athletes and hence the aim of this study was to demonstrate regional strain patterns in RT athletes using AAS.

**Methods:** Seventy-seven RT athletes (29 ± 4.9 (18–40 years) (84% males/16% females) engaged in > 4 h structured resistance exercise per/week were recruited into the study and grouped based on self-reported AAS user status: users (n = 57) and non-users (n = 20). Participants underwent assessment of body size and a full transthoracic echocardiogram to provide indices of LV structure (mean wall thickness (from basal and mid levels), LV end diastolic volume index (LVEDVi) and LVM index and function (ejection fraction and GLS). Regional longitudinal strain was presented from 17 segments from the base, mid and apex. Following assessment of normal distribution, all data was compared using a Students-Independent T-Test. A 2-way sample ANOVA was used to establish differences between base, mid and apical strain.

**Results:** All demographic data are presented in Table 1. AAS users had significantly greater LVM index (114.6 ± 32.5 vs 73.3 ± 13 g/m^2^, P < 0.001) and mean wall thickness (9.9 ± 1.4 vs 7.5 ± 0.7 mm, P < 0.001) compared to non-users but with no difference in LVEDVi. Ejection fraction (55 ± 6 vs 61 ± 4%, P < 0.001) and GLS (− 12.8 ± 5.3 vs − 17 ± 5.6%, P < 0.001) were significantly lower in users compared to non-users. Users had the lowest strain values from basal segments (− 11.9 ± 9.3 vs − 16.9 ± 6.2%, P = 0.016) compared to mid (− 14.2 ± 4.8 vs − 19.3 ± 3.9%, P = 0.010) and apical levels (− 16.6 ± 4.8 vs − 19.8 ± 3%, P = 0.007) (See Figure 1).

**Figure 1 (abstract ABS017):**
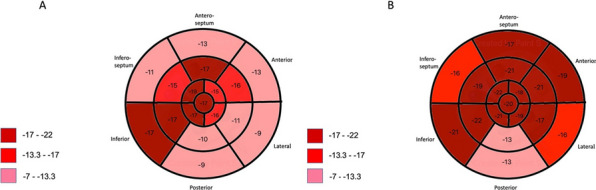
Regional longitudinal strain for each segment between A) current and B) non-users

**Conclusion:** RT individuals using AAS demonstrate increased LVM index and mean wall thickness with an associated reduction in systolic function when compared to non-users. Longitudinal strain values are reduced at all levels of the LV, with the largest reduction observed at the basal segments. This potentially represents the myocardial areas under greatest contractile stress.

**Table 1 (abstract ABS017):** Displaying demographic data across current and non-users, with P values to display significance

Variable	Non-user Mean ± SD	Current user Mean ± SD	P value
Sample size	20	57	
Age	27 ± 6	30 ± 4	0.006
Weight (kg)	76 ± 14	102 ± 18	< 0.001
Height (cm)	170 ± 9	178 ± 9	< 0.001
BSA (m^2^)	1.89 ± 0.22	2.24 ± 0.24	< 0.001
Heart rate (bpm)	56 ± 9	68 ± 11	< 0.001
Systolic BP (mmHg)	119 ± 11	127 ± 10	0.092
Diastolic BP (mmHg)	72 ± 10	74 ± 9	0.331
Training duration (years)	11 ± 9	12 ± 7	0.692
Training hours (per week)	9 ± 3	11 ± 3	0.193

## ABS018 Departmental compliance to national guidelines for the echocardiographic assessment of aortic stenosis

### Liam Rhys Davies^1^, Navroz Masani^1^

#### ^1^University Hospital of Wales, Cardiff, UK

*Echo Research & Practice* 2024, **11(Suppl 1)**:ABS018

**Background:** Aortic stenosis (AS) is a complex valve disease, which has a significant influence on cardiovascular related morbidity and mortality. Transthoracic echocardiography is the most widely accessible clinical diagnostic tool used to assess its severity and the measurements obtained are highly influential in the subsequent clinical decision-making. Therefore, accurate, reliable and reproducible measurements are critical.

**Purpose:** To assess the University Hospital of Wales’ (UHW) assessment of AS by measuring their compliance to the British Society of Echocardiography’s guideline.

**Methods:** Data from 118 patients were retrospectively analysed. Performance of the essential images and measurements were compared to guideline instructions and scored accordingly. A score of “0” was given for a poor/non-existent image/measurement. A score of “1” was awarded for good quality and compliance to most of the guideline instructions. Finally, a score of “2” highlighted a perfect performance and compliance to the guideline instructions; with full optimisation and no error.

**Results:** The left ventricular outflow tract diameter (LVOT) measurement scored perfect in 67 studies (59%). Tracing of the LVOT pulsed-wave Doppler envelope scored good in 61 studies (52%). Continuous-wave aortic Doppler envelope tracing scored perfect in 64 studies (54%). The two-dimensional parasternal long axis image scored perfect in 108 studies (92%). The apical-three chamber pulsed-wave Doppler image scored poor in 108 studies (92%). PEDOFF probe imaging scored poorly in 60 studies (51%).

**Conclusion:** Overall, UHW’s assessment of AS is very good. However, there are areas of assessment which do require addressing and may require further teaching and training.

## ABS020 Reproducibility of three-dimensional echocardiographic derived left ventricular structure, function and myocardial strain in young healthy individuals

### Christopher Johnson^1^, Robert M. Cooper^1^, Keith George^1^, Martin Stout^2^, David Oxborough^1^

#### ^1^Research Institute for Sport and Exercise Sciences, Liverpool John Moores University, Liverpool, UK; ^2^School of Healthcare Science, Manchester Metropolitan University, Manchester, UK

*Echo Research & Practice* 2024, **11(Suppl 1)**:ABS020

**Background:** Three-dimensional (3D) transthoracic echocardiography (TTE) allows for the quantification of left ventricular (LV) structure, function and myocardial strain. The clinical adoption of this technology requires demonstration of best-case, real-world (repeated acquisition) reproducibility. In view of this, the aim of the current study was to determine the intra- and inter-observer reproducibility of 3D derived LV structural, functional and myocardial strain parameters in young healthy volunteers.

**Methods:** Thirteen healthy controls (24 ± 3 years; n = 6 male; n = 7 female) were recruited (see Table 1). Participants were required to attend two separate visits to the laboratory (3–5 days apart at the same time of day; see Figure 1). A single sonographer acquired and analysed a 3D TTE on both visits (intra-observer). A second sonographer acquired and analysed a further 3D TTE on the first visit (inter-observer). Participant height, body mass, blood pressure and an electrocardiogram were recorded at each visit. LV volumes, mass, ejection fraction, global longitudinal, radial, circumferential, area strain and twist were measured. Reproducibility was assessed using a Paired T-Test, intraclass correlation coefficients (ICC) and coefficients of variation (CoV).

**Results:** There was no evidence of systematic bias as determined by paired T-tests for any parameter. Intra- and inter-observer reproducibility of 3D structure and function was generally “moderate” to “excellent” (ICC 0.45–0.95 and 0.40–0.81 respectively) with relatively small CoV (4–14% and 6–14% respectively; see Table 2). Sphericity index demonstrated “poor” intra- and inter-observer reproducibility (ICC − 0.13 and − 0.10 respectively) with relatively large CoV (16% and 18% respectively). Intra- and inter-observer reproducibility of 3D myocardial strain parameters were generally “moderate” to “good” (ICC 0.50–0.60 and 0.48–0.78 respectively) with relatively small CoV (7–10% and 6–9% respectively). Twist demonstrated “poor” intra-observer and “moderate” inter-observer reproducibility (ICC 0.35 and 0.48 respectively) with relatively large CoV (50% and 35% respectively).

**Conclusion:** 3D TTE derived structure, function and myocardial strain demonstrates good intra- and inter-observer reproducibility in a best-case setting i.e., young healthy individuals. This is with exception of sphericity index and twist which demonstrated consistently low reproducibility. Our findings, along with the recognised benefits of 3D TTE, suggest there is potential for the adoption of this technology in regular clinical imaging. However, evidence of 3D TTE reproducibility needs to be replicated in larger more diverse and diseased populations. Furthermore, the standardisation of 3D TTE acquisition and analysis is essential for safe adoption of this technology in clinical practice.

**Figure 1 (abstract ABS020):**
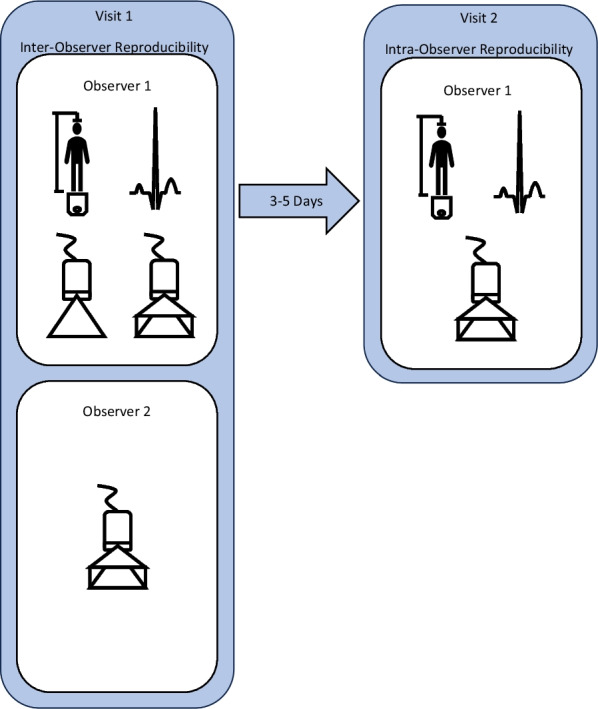
Protocol schematic. Visit 1—observer 1 and 2 acquired and analysed a 3D TTE to assess inter-observer reproducibility. Visit 2—observer 1 acquired and analysed a second 3D TTE to assess intra-observer reproducibility

**Table 1 (abstract ABS020):** Participant demographics and conventional two-dimensional echocardiography

	Visit 1 Mean ± SD	Visit 2 Mean ± SD
*Participant demographics*		
Sex (male/female)	6/7	6/7
Age (y)	24 ± 3	24 ± 3
Height (cm)	171 ± 8	171 ± 8
Body mass (kg)	70 ± 8	70 ± 8
Body surface area (m^2^)	1.83 ± 0.14	1.83 ± 0.14
Systolic BP (mmHg)	117 ± 8	117 ± 8
Diastolic BP (mmHg)	67 ± 9	68 ± 9

BP: blood pressure

**Table 2 (abstract ABS020):** Intra- and inter-observer reproducibility of three-dimensional left ventricular structure, function and myocardial strain

	Analysis 1 (mean ± SD)	Analysis 2 (mean ± SD)	ICC (95% CI)	CoV (%)	One-Way ANOVA (Bonferroni)
*Intra-observer reproducibility*					
End-diastolic volume (ml)	134 ± 23	132 ± 26	0.95 (0.86–0.99, P < 0.001)	4	P = 1.0
End-systolic volume (ml)	54 ± 10	53 ± 11	0.88 (0.67–0.96, P < 0.001)	7	P = 1.0
Ejection fraction (%)	60 ± 4	59 ± 5	0.60 (0.07–0.86, P = 0.016)	5	P = 1.0
Heart rate (beats/min)	60 ± 12	61 ± 10	0.61 (0.09–0.86, P = 0.014)	11	P = 1.0
Stroke volume (ml)	80 ± 15	79 ± 19	0.90 (0.71–0.97, P < 0.001)	7	P = 1.0
Cardiac output (L/min)	4.7 ± 0.6	4.7 ± 1.1	0.45 (− 0.14–0.80, P = 0.061)	14	P = 1.0
Sphericity index	0.40 ± 0.08	0.42 ± 0.05	− 0.13 (− 0.68–0.45, P = 0.663)	16	P = 1.0
End-diastolic mass (g)	134 ± 21	137 ± 22	0.89 (0.69–0.97, P < 0.001)	5	P = 1.0
Global longitudinal strain (%)	− 15.9 ± 1.9	− 16.0 ± 2.2	0.50 (− 0.08–0.82, P = 0.042)	9	P = 1.0
Twist (°)	8.3 ± 4.4	6.4 ± 4.5	0.35 (− 0.18–0.74, P = 0.103)	50	P = 0.926
Global circumferential strain (%)	− 15.4 ± 2.2	− 15.1 ± 2.1	0.60 (0.10–0.86, P = 0.013)	9	P = 1.0
Global radial strain (%)	42.6 ± 4.7	41.4 ± 6.9	0.51 (− 0.03–0.82, P = 0.034)	10	P = 1.0
Global area strain (%)	− 27.9 ± 2.6	− 27.6 ± 3.3	0.51 (0.05–0.82, P = 0.036)	7	P = 1.0
*Inter-observer reproducibility*					
End-diastolic volume (ml)	134 ± 23	118 ± 27	0.74 (− 0.05–0.93, P < 0.001)	11	P = 0.646
End-systolic volume (ml)	54 ± 10	47 ± 14	0.73 (0.05–0.93, P < 0.001)	14	P = 0.768
Ejection fraction (%)	60 ± 4	61 ± 5	0.40 (− 0.16–0.77, P = 0.08)	6	P = 1.0
Heart rate (beats/min)	60 ± 12	62 ± 12	0.91 (0.75–0.97, P < 0.001)	6	P = 1.0
Stroke volume (ml)	80 ± 15	71 ± 15	0.63 (0.08–0.88, P = 0.001)	13	P = 1.0
Cardiac output (L/min)	4.7 ± 0.6	4.3 ± 0.4	0.33 (− 0.13–0.72, P = 0.075)	11	P = 1.0
Sphericity index	0.40 ± 0.08	0.40 ± 0.07	− 0.10 (− 0.68–0.48, P = 0.623)	18	P = 1.0
End-diastolic mass (g)	134 ± 21	129 ± 24	0.81 (0.49–0.94, P < 0.001)	8	P = 1.0
Global longitudinal strain (%)	− 15.9 ± 1.9	− 15.7 ± 2.1	0.78 (0.42–0.93, P = 0.001)	6	P = 1.0
Twist (°)	8.3 ± 4.4	9.7 ± 4.2	0.48 (− 0.04–0.81, P = 0.038)	35	P = 1.0
Global circumferential strain (%)	− 15.4 ± 2.2	− 16.0 ± 1.9	0.54 (0.03–0.83, P = 0.022)	9	P = 1.0
Global radial strain (%)	42.6 ± 4.7	42.7 ± 4.9	0.53 (− 0.03–0.83, P < 0.030)	8	P = 1.0
Global area strain (%)	− 27.9 ± 2.6	− 28.3 ± 2.3	0.48 (− 0.08–0.81, P = 0.045)	6	P = 1.0

CI: confidence interval; CoV: coefficient of variation; ICC: intraclass correlation; SD: standard deviation

## ABS022 Three-dimensional transthoracic echocardiography—current clinical practice and future direction—“Is now the right time?”

### Liam Corbett^1^, Patrick O'Driscoll^1^, Elena Surkova^2,3^

#### ^1^Liverpool Heart and Chest Hospital NHS Foundation Trust, Liverpool, UK; ^2^Royal Brompton and Harefield Hospitals, Guy’s and St. Thomas’ NHS Foundation Trust, London, UK; ^3^National Heart and Lung Institute, Imperial College London, UK

*Echo Research & Practice* 2024, **11(Suppl 1)**:ABS022

**Background:** Three-dimensional echocardiography (3DE) imaging has been a major advance in left ventricular (LV) and right ventricular (RV) size and function quantification. We evaluated the use of current clinical standards for the quantification of LV and RV function, training in 3DE, and aimed to ascertain barriers preventing the practice of 3DE quantification. Through the British Society of Echocardiography (BSE) regional rep network, echocardiographers were invited to participate in an open online survey. A total of 181 participants from UK echocardiography departments, mostly from tertiary centres (61%) completed the 28-question survey. For 3DE quantification, 3DE-LV was adopted more frequently than 3DE-RV (48% vs 11%, respectively). Imaging feasibility was a recognised factor in 3DE RV and LV adoption (30% and 50%, respectively). While 93% of respondents had access to 3D probes, the largest barrier to routine adoption was time, with 68% reporting less than the BSE standard of 45–60 min (8% < 30-min). Training deficiencies in 3DE were highlighted, with 83% reporting they would like further formal training opportunities. Furthermore, of those respondents who had undertaken professional accreditation, 89% were not formally assessed for 3DE assessment. This is additionally likely to be mirrored in societies departmental accreditation packages. This UK survey also reported good accessibility to magnetic resonance imaging (72%), which was reported to impact overall 3DE adoption. Although advanced parameters such as 3DE are now readily available, it remains underutilised. More formal training, assessment, improved adoption of BSE scanning times, alongside industry and societies support should increase utilisation.

